# Effects of Metformin Added to Insulin in Adolescents with Type 1 Diabetes: An Exploratory Crossover Randomized Trial

**DOI:** 10.1155/2020/7419345

**Published:** 2020-12-24

**Authors:** Daizhi Yang, Jinhua Yan, Hongrong Deng, Xubin Yang, Sihui Luo, Xueying Zheng, Jing Lv, Wen Liang, Mengjie Hong, Zekai Wu, Bin Yao, Jianping Weng, Wen Xu

**Affiliations:** ^1^Department of Endocrinology and Metabolism, The Third Affiliated Hospital of Sun Yat-sen University, Guangzhou 510630, China; ^2^Guangdong Provincial Key Laboratory of Diabetology, Guangzhou 510630, China; ^3^Department of Endocrinology and Metabolism, The First Affiliated Hospital of USTC, Division of Life Sciences of Medicine, University of Science and Technology of China, Anhui 230026, China; ^4^Department of Endocrinology and Metabolism, The Fifth Affiliated Hospital of Sun Yat-sen University, Zhuhai 519000, China; ^5^Department of Cardiovascular Medicine, The Seventh Affiliated Hospital of Sun Yat-sen University, Shenzhen 518107, China

## Abstract

**Background:**

To comprehensively assess the effects of metformin added to insulin on metabolic control, insulin sensitivity, and cardiovascular autonomic function in adolescents with type 1 diabetes.

**Materials and Methods:**

This was an exploratory, crossover, randomized trial conducted in adolescents with type 1 diabetes aged 12-18 years old. Participants were randomly received metformin (≤1000 mg/d) added to insulin for 24 weeks followed by insulin monotherapy for a subsequent 24 weeks or vice versa. Blood pressure, body mass index, insulin dose, estimated insulin sensitivity, glycated hemoglobin A1c (HbA1c), and lipid profiles were measured, with a 72-hour continuous glucose monitoring and 24-hour Holter monitoring performed at baseline, 24, and 50 weeks for the assessments of glucose variability and heart rate variability.

**Results:**

Seventeen patients with mean ± SD age 14.4 ± 2.3 years, body mass index 18.17 ± 1.81 kg/m^2^, median (IQR) diabetes duration 4.50 (3.58, 6.92) years, and HbA1c 9.0% (8.5%, 9.4%) were enrolled. The between-group difference in HbA1c of 0.28% (95% CI -0.39 to 0.95%) was not significant (*P* = 0.40). Changes in body mass index, insulin dose, blood pressure, lipid profiles, and estimated insulin sensitivity were similar for metformin add-on vs. insulin monotherapy. Glucose variability also did not differ. Compared with insulin monotherapy, metformin add-on significantly increased multiple heart rate variability parameters.

**Conclusions:**

Metformin added to insulin did not improve metabolic control or glucose variability in lean/normal-weight adolescents with type 1 diabetes. However, metformin added to insulin significantly increased heart rate variability, suggesting that metformin might improve cardiovascular autonomic function in this population.

## 1. Introduction

The incidence of childhood-onset type 1 diabetes (T1D) is increasing worldwide [1, 2]. Patients with childhood-onset T1D have a lifetime exposure of hyperglycemia which contributes to increase risk for premature diabetes-related complications, including cardiovascular disease (CVD). The Diabetes Control and Complications Trial (DCCT) demonstrated that intensive glycemic control in T1D reduced diabetes complications [3]. However, the DCCT also showed that adolescents with T1D had higher glycated hemoglobin A1c (HbA1c) level than adult patients, despite a greater daily insulin requirement and weight gain, suggesting insulin administration was less effective in maintaining glycemic control in the adolescent cohort, partially due to insulin resistance in puberty [4]. The increase in puberty-related hormones including growth hormone and sex steroids may contribute to insulin resistance in adolescents [5, 6]. Hence, strategies which could improve insulin sensitivity should be considered for better diabetes management in adolescent patients with T1D.

Metformin acts primarily to decrease hepatic glucose output, increase peripheral glucose uptake and utilization, and thus improve insulin sensitivity. Metformin is allowed to be prescribed to patients over 10 years old and to patients with T1D in the instruction. However, metformin is not routinely used in daily clinical practice, although the treatment of metformin added to insulin in adolescents with T1D has been increasingly investigated [7, 8]. Previous studies revealed that metformin did not improve glycemic control but decreased insulin dose and measures of adiposity and improved insulin resistance in overweight/obese adolescents as well as normal-weight adolescents with T1D [9–11]. However, these studies were conducted among Caucasian patients with T1D. Compared with Caucasian patients, patients with T1D in China are characterized with a much lower median body mass index (BMI) of around 19.6 kg/m^2^ [12]. Whether metformin can improve glycemic control and other metabolic disturbances in this relatively low-BMI population remains to be explored.

T1D increases the risk of CVD 4- to 8-fold [13]. Moreover, insulin resistance has been increasingly recognized to play an important role in the pathophysiology of CVD in patients with T1D [14–16]. On the other hand, abnormal cardiovascular autonomic function characterized by sympathovagal imbalance and subsequent impaired heart rate regulation is closely associated with cardiovascular morbidity and mortality in diabetes [17, 18]. It is a serious but overlooked complication of diabetes, especially in youth, because it is mostly asymptomatic in early stage but ultimately leads to cardiovascular complications. In patients with type 2 diabetes, metformin treatment is associated with improvements in cardiac sympathovagal balance [19]. Although several randomized trials have reported effects of metformin treatment on metabolic parameters and insulin resistance in adolescents with T1D [9–11], impacts of metformin on cardiovascular autonomic function in this population have not yet been comprehensively investigated.

Therefore, this study is aimed at examining the effects of metformin added to insulin on metabolic control and insulin sensitivity, as well as the cardiovascular autonomic function represented by heart rate variability (HRV), in Chinese adolescents with T1D.

## 2. Materials and Methods

This was a 50-week, randomized, crossover, single-center clinical trial in youth with T1D. The protocol was approved by the Institutional Review Board of the Third Affiliated Hospital of Sun Yat-sen University, and all procedures were conducted at this hospital. Written informed consents were obtained from participants or guardians as appropriate for age. The trial was registered at Clinicaltrials.gov (NCT02765347).

### 2.1. Participants

Inclusion criteria were patients with T1D with diabetes duration ≥ 1 year, age of 12 to 18 years, Tanner stage of 2 to 5, and HbA1c level of 7.5% to 10%, treated with either an insulin pump or multiple daily injections of insulin for at least 6 months; total daily insulin dose (TDD) ≥ 0.8 units/kg per day and the dose was stable for at least 1 month (TDD variation < 10%) before enrollment. The diagnosis of T1D was established by an endocrinologist. And the patients must be insulin dependent at or shortly after diagnosis and were tested positive for one or more T1D-associated autoantibodies. Major exclusion criteria included history of ≥1 diabetic ketoacidosis events in the past 3 months, frequent episodes of severe hypoglycemia, hepatic function impairment (alanine aminotransferase (ALT) ≥ 2.5 times higher than the upper limit of normal), moderate to severe renal impairment (estimated glomerular filtration rate (eGFR) < 60 mL/min/1.73m^2^, calculated from MDRD equation), clinically significant stage of cardiac disease, pregnancy, and use of medications affecting insulin sensitivity (oral steroids, immunosuppressants, etc.) or HRV (*β* blockers, etc.) within 60 days.

### 2.2. Study Design

**Figure 1:**
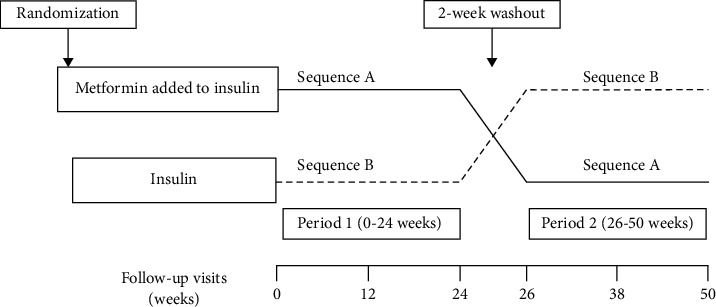
Eligible participants were randomized 1 : 1 to receive metformin (started at a dose of 500 mg daily for 1 week, with a subsequent dose escalation to 500 mg twice daily) added to insulin for 24 weeks followed by insulin treatment alone for a subsequent 24 weeks (sequence A) or vice versa (sequence B), separated by a 2-week washout period. The face-to-face followed-up visits were at weeks 0, 12, 24, 26, 38, and 50.

A randomization schedule was generated by IBM SPSS Statistics, version 23 (IBM Corp., Armonk, NY, USA). And the randomization was performed using sealed envelopes. Eligible participants were randomized 1 : 1 to receive metformin (1000 mg/d) added to insulin for 24 weeks followed by insulin treatment alone for a subsequent 24 weeks (sequence A) or vice versa (sequence B), separated by a 2-week washout period (Figure 1). The metformin started at a dose of 500 mg daily for 1 week, with a subsequent dose escalation to 500 mg twice daily (1000 mg daily). Participants' insulin doses were adjusted based on the following goal range: before-meal blood glucose between 5.0 and 7.2 mmol/L and bedtime/overnight blood glucose between 5.0 and 8.3 mmol/L [20]. Participants received instructions on medical nutrition therapy at enrollment. The face-to-face followed-up visits were at weeks 0, 12, 24, 26, 38, and 50 to collect patients' information and provide guidance. Participants were asked to perform 7-point self-monitoring blood glucose at least in two consecutive days before each visit.

At baseline, each participant's height, weight, blood pressure, and waist circumference were measured. Fasting blood samples were drawn for measurements of HbA1c, lipid profiles (total cholesterol (TC), high-density lipoprotein cholesterol (HDL-C), low-density lipoprotein cholesterol (LDL-C), and triglyceride (TG)), liver enzymes (ALT and aspartate aminotransferase (AST)), and creatinine. Mixed-meal tests were performed. Fasting and 2-hour postprandial serum C-peptide levels were measured during mixed-meal tests. After these measurements, each participant underwent a comprehensive baseline evaluation of glucose variability and cardiovascular autonomic function assessed by a 72 h continuous glucose monitoring (CGM) and 24-hour Holter monitoring, respectively. After each 24-week treatment period, all participants underwent the repeated study assessments identical to the baseline assessments. Namely, all these above measurements were performed at baseline, 24 weeks, and 50 weeks.

### 2.3. Assessments of Insulin Sensitivity

Glucose disposal rate derived from a euglycemic-hyperinsulinemic clamping test is considered the gold standard to estimate insulin sensitivity. Considering the complicated procedure of a euglycemic-hyperinsulinemic clamping test, insulin sensitivity in this study was assessed by lnIS (calculated by the equation from the SEARCH study: lnIS = 4.64725 − 0.02032 × waist (cm) − 0.09779 × A1c (%) − 0.00235 × TG (mg/dL, to convert TG values from mmol/L to mg/dL, divide by 0.0113)), which has been validated using the euglycemic-hyperinsulinemic clamp testing in the SEARCH study [21].

### 2.4. Assessments of Glucose Variability

Each participant was asked to wear a retrospective continuous glucose monitor (CGM) sensor (iPro2 digital recorder, Medtronic Diabetes) for 72 hours at baseline and the end of each crossover period (24 weeks and 50 weeks). Parameters representing glucose variability were obtained from CGM system, including (1) coefficient of variability of glycemia (% CV: standard deviation of blood glucose (SDBG)/mean blood glucose (MBG) × 100), (2) the mean amplitude of glycemic excursions (MAGE): the arithmetic mean of the differences between consecutive peaks and nadirs with measurement in the peak-to-nadir direction by the first qualifying excursion, and (3) absolute means of daily differences (MODD): the mean of absolute differences between glucose values at the same time on two consecutive days.

### 2.5. Assessments of Cardiovascular Autonomic Function

The HRV representing cardiovascular autonomic function was measured by using a 24-hour ambulatory electrocardiograph monitoring (Marquette, USA). Participants were instructed to avoid caffeine, alcohol, cigarette, and heavy exercise for at least 24 hours before the tests. The indices of HRV adopted in this study included time domain and frequency domain indices. Time domain indices were (1) the mean of the 5-minute standard deviations of RR intervals calculated over 24 hours (SDNN, ms), (2) the standard deviation of the average RR intervals calculated over 5 minutes (SDANN, ms), (3) the percentage of the interval differences of successive RR intervals greater than 50 ms (PNN50, %), and (4) the square root of the mean squared differences of successive RR intervals (RMSSD, ms). Frequency domain indices were low frequency band power (LF, range 0.04-0.15 Hz), high frequency band power (HF, range 0.15-0.4 Hz), and ratio of LF and HF (LF/HF).

### 2.6. Study Outcomes

The primary outcome was the change in HbA1c from baseline to the end of the entire study. Exploratory endpoints included changes in BMI, blood pressure, serum lipid profiles, insulin sensitivity, total daily insulin per kg of body weight, glycemic variability parameters obtained from CGM system, and HRV representing the cardiovascular autonomic function.

Patient's compliance was monitored based on the pill counts at each study visit. All reported adverse events from baseline to the end of the study were reported regardless of whether the events were considered treatment-related or not. The safety outcomes included gastrointestinal events, hypoglycemia, severe hypoglycemia, diabetic ketoacidosis, and lactic acidosis. Self-reported hypoglycemic episodes were classified according to the American Diabetes Association (ADA) criteria: glucose alert value (level 1) is defined as a measurable glucose concentration of ≤3.9 mmol/L but ≥3.0 mmol/L; clinically significant hypoglycemia (level 2) is defined as a measurable glucose concentration < 3.0 mmol/L, and severe hypoglycemia (level 3) is defined as severe cognitive impairment requiring external assistance for recovery [22].

### 2.7. Statistical Analysis

We did not conduct an a priori power analysis as this study was mainly an exploratory study. Data are presented as the mean ± standard deviation (SD) or median (interquartile range (IQR)) for continuous variables and *n* (%) for categorical variables. Prior to all analyses, normality was examined by the Shapiro Wilks test. Baseline differences between treatment sequence groups were tested using *t*-test, chi-squared test or Fisher's exact test, or Mann–Whitney *U* test when appropriate. Endpoint analyses about metabolic parameters such as HbA1c, blood pressure, lipid profiles, BMI, and TDD were conducted in the per-protocol population, as well as in the intention-to-treat population for supporting analysis. Endpoint analyses about glucose variability and HRV indexes were performed in patients with valid recordings for a minimum of one period. Safety analyses were conducted in the intention-to-treat population, which included all randomly assigned patients.

For normally distributed values, linear mixed effects models were used, with treatment arms (insulin alone or metformin added to insulin), treatment sequence (sequence A or B), carryover effects, and study period (period 1 or 2) as fixed effects and study participants and study period as nested random effects. HRV indexes which did not meet normality assumptions (LF and HF) were transformed as appropriate. For other nonnormally distributed values, the Wilcoxon signed rank test was used for pairwise comparisons. For binary data, the McNemar test was used for comparison of repeated measurements. The association between variables of interest was examined by using multiple linear regression analysis. A *P* value < 0.05 was considered statistically significant. Statistical analyses were carried out using Stata SE version 14.0 (StataCorp LP, College Station, Texas) and IBM SPSS Statistics, version 23 (IBM Corp., Armonk, NY, USA).

## 3. Results

**Figure 2:**
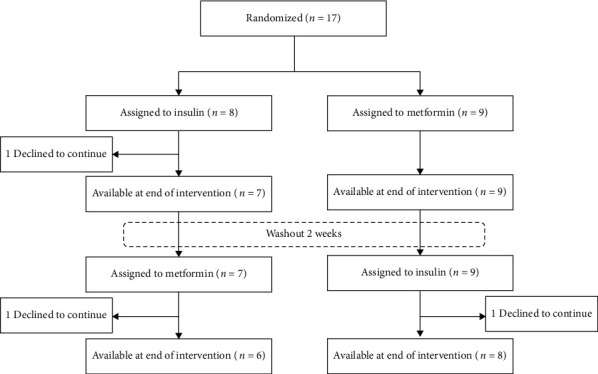
CONSORT (Consolidated Standards of Reporting Trials) flow diagram.

Of 63 screened participants, 17 were subsequently randomly assigned, with 9 allocated to sequence A (metformin to nonmetformin) and 8 to sequence B (nonmetformin to metformin). Of these 17 patients, 3 withdrew due to long distance to the follow-up site, and among these 3 patients, 1 withdrew during the first period, and 2 withdrew during the second period (Figure 2). The characteristics of the 2 sequence groups at randomization were well balanced (Table 1). Across the sequence groups, 7 were female (41.2%), mean age was 14.4 ± 2.3 years, median diabetes duration was 4.50 years (IQR 3.58-6.92), HbA1c was 9.0% (8.5-9.4), and systolic and diastolic blood pressure were 109.4 ± 10.5 mmHg and 64.5 ± 6.4 mmHg, respectively. The insulin dose was 0.94 (0.89-1.02) units per kg body weight per day, lnIS was 2.24 ± 0.18, and TC, LDL-C, HDL-C, and G were 4.55 ± 1.15 mmol, 2.53 mmol/L (2.13-3.25), 1.64 ± 0.36 mmol/L, and 0.61 mmol/L (0.51-0.76), respectively. The baseline mean BMI was 18.17 ± 1.81 kg/m^2^, ranging from 15.22 to 21.72 kg/m^2^.

At the end of the study, only one participant failed to titrate to a metformin dose of 1000 mg/d. The others maintained the metformin dose of 1000 mg/d. Changes in metabolic parameters, glucose variability, and HRV with each treatment period from baseline are shown in Table 2. With the 24-week interventions of either metformin added to insulin or insulin alone treatment, mean change in HbA1c from baseline was 0.25% in the metformin group and 0.03% in the nonmetformin group (estimated between group difference, 0.28% (95% CI, −0.39% to 0.95%); *P* = 0.40). There were no significant changes in HbA1c in both the metformin and nonmetformin groups at each study time point (12 weeks and 24 weeks during each study period) compared to baseline HbA1c (data not shown). Changes in systolic and diastolic blood pressure and blood lipid profiles were similar between metformin and nonmetformin groups. There were no differences regarding the changes in BMI, daily insulin dose, and insulin sensitivity expressed as lnIS between groups. Changes in MBG and glucose variability indexes including %CV, SDBG, MAGE, and MODD were not significantly different between groups as well (all *P* > 0.05).

Analysis of the 24-hour time domain parameters of HRV revealed that metformin add-on treatment significantly increased the SDNN compared with insulin treatment alone (19.5 ± 31.1 vs. −4.8 ± 20.8 ms; between group difference, 26.96 ms [95% CI 2.24 to 51.69]; P =0.03) (Table 2). For SDANN and PNN50, significant increases were also observed in the metformin group compared with the nonmetformin group (difference, 25.62 ms (0.15 to 51.09), *P* = 0.049, and 10.14 ms (3.74 to 16.55), *P* = 0.004, respectively). The significant associations between metformin treatment and changes in SDNN (*β* = 24.3; *P* = 0.04), SDANN (*β* = 25.6; *P* = 0.03), and PNN50 ms (*β* = 9.3; *P* < 0.01) were unchanged after adjustment for changes in HbA1c, systolic blood pressure, and MAGE in a multivariable analysis (*β* = 28.6, 31.0, and 9.6, respectively; all *P* < 0.05), suggesting the independent increase in SDNN, SDANN, and PNN50 by metformin add-on therapy (Table 3).

Analysis of frequency domain parameters showed metformin add-on treatment significantly increased the log-transformed values of HF power compared with insulin treatment alone (0.01 ± 0.35 vs. −0.19 ± 0.34 ms^−1^, difference, 0.38 ms^−1^ (0.09 to 0.66), *P* = 0.01), with a significant decrease in the LF/HF ratio in the metformin group compared with that in the nonmetformin group (−0.27 ± 0.26 vs. 0.16 ± 0.36, difference, -0.47 (-0.75, -0.18), *P* = 0.006). Change in LogLF was similar between the two groups (Table 2).

No severe hypoglycemia or other severe adverse events were reported in both groups during the study. Level 1 hypoglycemia was reported in 8 subjects (47.1%) in the metformin group and 10 subjects (58.8%) in the nonmetformin group (*P* = 0.63), while level 2 hypoglycemia occurred in 2 subjects in both groups (*P* = 1.0). Gastrointestinal side effects were limited to nausea, abdominal pain, and diarrhea, which were reported in 3 participants in the metformin group (17.6%) compared with 1 participant in the nonmetformin group (5.9%; *P* < 0.01). No participants from the metformin group were excluded because of these symptoms. There were no significant changes in hemoglobin, ALT, AST, or serum creatinine at the end of the study compared with the relevant baseline values in both treatment groups (data not shown).

## 4. Discussion

The primary finding of this current randomized, crossover, controlled clinical trial is that metformin added to insulin did not improve metabolic control including blood glucose, blood pressure, lipid profiles, and body weight, as well as glucose variability as measured with parameters obtained from CGM and insulin sensitivity as measured with lnIS among lean/normal-weight adolescents with T1D inadequately controlled with insulin. And the insulin dosages of these patients were not reduced after the addition of metformin. There have been only a few studies examining the use of metformin in such specific patient group [10, 11]. In addition, this study found that metformin improved HRV despite no obvious effects on metabolic parameters, suggesting the potential benefits of metformin on cardiovascular autonomic function. To the best of our knowledge, this is the first report demonstrating improvement in 24-hour HRV by Holter monitoring with metformin add-on therapy in adolescents with T1D.

The most recent meta-analysis showed that metformin was not associated with glycemic control in T1D patients, although it exhibited other benefits, such as lower BMI and reduced insulin requirements [23]. Similarly, most of the randomized trials and meta-analysis evaluating the effect of metformin add-on in adolescents with T1D found that metformin reduced the total insulin dose and BMI, suggesting improvements in insulin resistance, but did not improve HbA1c [9–11, 24, 25]. In this study, we found no improvement in HbA1c with metformin, which was consistent with previous studies. However, we did not observed improvements in total insulin dose, insulin sensitivity (represented by lnIS), or BMI. There are several possible explanations for these results. First, it should be noted that the participants' BMI level in our study (baseline mean BMI 18.17 kg/m^2^, ranging from 15.22 to 21.72 kg/m^2^) is much lower than those reported in the previous studies [9–11]. It is assumed that metformin add-on therapy might not be able to decrease BMI on the basis of such a low baseline BMI level in our study. Second, in Bjornstad's study, insulin sensitivity was evaluated by the gold standard multiphase hyperinsulinemic euglycemic clamping test, and metformin was found to improve insulin resistance regardless of baseline BMI, weight, fat mass, and insulin dose [11]. Insulin resistance in this study was assessed by lnIS. Although lnIS is considered a good index of insulin resistance and convenient to be obtained with clinical variables [21, 26], it may not be sensitive enough to examine the effect of metformin on insulin sensitivity in this population. Third, considering the low BMI of our participants, metformin dose in this study was titrated to 1000 mg per day by the investigators, which was smaller than 1500-2000 mg per day in most of the previous studies. The lower dose of metformin using in this study may also partly lead to the discrepancy. Lastly, the relatively small sample size due to the pilot design of the study might have affected the power to examine the effects of metformin on various metabolic measurements, even with the crossover design and relatively long follow-up period.

Regarding other metabolic parameters, this study did not prove significant improvements in blood pressure and lipid profiles with metformin treatment. Glucose variability parameters (CV%, MAGE, and MODD) were all similar between the metformin add-on treatment and insulin treatment alone. These results were in line with the findings of Libman's and Pitocco's studies [9, 27]. On the contrary, among patients with type 2 diabetes treated with insulin pump or multiple daily injections of insulin, metformin add-on therapy was associated with improvement in glucose fluctuation and reduced risk of hypoglycemia [28]. The reasons caused the different effect of metformin on glucose variability among patients with type 1 or type 2 diabetes remained unclear.

Cardiovascular autonomic neuropathy subclinically presented in 20–36% of patients with T1D [29]. Specifically, among adolescents and young adults with T1D, the prevalence of cardiovascular autonomic neuropathy was 12% [30]. Previous studies found that HRV were significantly reduced in patients with T1D versus healthy controls [31–33]. Reduced HRV is known to indicate poorer autonomic function and has been recognized as an early predictor of cardiovascular autonomic neuropathy [34, 35]. That is why we chose HRV to represent cardiovascular autonomic function in this study. In overweight patients with type 2 diabetes, a randomized controlled trial documented that metformin treatment was associated with a significant improvement in cardiac sympathovagal balance, and this beneficial effect was associated with decreased plasma free fatty acids and insulin resistance [19]. However, in T1D, an observational study included 42 patients with T1D treated with insulin plus metformin and 84 matched participants. This study found no association of metformin use and improvements in SDNN or RMSSD [36]. On the contrary, in the current crossover, randomized trial, we observed increases in both HRV time domain indices (SDNN, SDANN, and PNN50) and frequency domain index (LogHF and LF/HF), which may be taken to indicate improvements in autonomic nervous system, despite no significant change in various metabolic parameters including HbA1c, blood pressure, lipid profiles, and body weight after metformin added to insulin. The SDNN, SDANN, PNNN50, and HF power reflect alteration in autonomic function that are primarily mediated by vagal activity, while LF power is modulated by both the parasympathetic and the sympathetic nervous systems [37, 38]. Taken together, the increase in SDNN, SDANN, PNN50, and LogHF may be contributed to improvement in vagal activity after metformin add-on therapy. Furthermore, multivariate linear regression analysis revealed that the increase in SDNN, SDANN, and PNN50 was unaffected by changes in HbA1c, systolic blood pressure, and glucose variability as assessed with MAGE. Metformin's effects on the heart and the cardiovascular system have been shown in many conditions [39]. In animal models, the cardiac autonomic effects of metformin had been proved to be along with its anti-inflammatory and antioxidant profiles and unrelated to its effects on glucose regulation [40]. In view of the pilot design of the current study and as the secondary endpoint, this result should be interpreted with caution. However, these findings suggested that metformin might have beneficial effects on the autonomic nervous system that is not mediated by an improvement in metabolic parameters. This possibility requires to be investigated in appropriately designed outcome trials in the future.

Our study has several strengths. First, it was the first crossover, randomized control trial of metformin add-on treatment in lean/normal-weight adolescents with T1D with comprehensive assessments including metabolic parameters, insulin sensitivity, glucose variability as assessed by CGM, and change in cardiovascular autonomic function as assessed by HRV derived from 24-hour Holter monitoring. Second, the randomized crossover design compensated for interindividual variability in insulin sensitivity, diet, and exercise patterns.

The relatively small sample size due to the pilot study design is one of the limitations. However, this enabled us to calculate a reasonable sample size which would allow an adequately powered examination of relevant study endpoints for future work. Post hoc power calculation based on actual SDNN data from the current study revealed that a sample size of 27 participants was required to achieve 80% power to detect a difference of 25 ms in SDNN measurements between metformin and nonmetformin groups, for a two-tailed *α* < 0.05. In fact, we are now conducting a multicentered, placebo-controlled, randomized clinical trial with larger sample size to verify the results from this study, registered at http://www.chictr.org.cn (ChiCTR1800019438). In addition, despite with recommendations on glucose target, no enforcement of dose titration was required in this study. Nevertheless, we considered this limitation would be expected to affect the results in both groups to a similar degree because of the randomized crossover design. Moreover, the dose of metformin used in this study was considered relatively small (1000 mg per day). Actually, our team recently revealed that in Chinese real-word clinical practice, the average dose of metformin added to adult patients with T1D is just 1000 mg per day [9]. In that case, this study reflects the real-world clinical practice in some degree. Finally, although we did not restrict the BMI in the inclusion criteria, participants enrolled in this study were all lean or normal-weight adolescents, which represented a specific patient group. Thus, results of this study could not be extrapolated to other patient population.

## 5. Conclusions

In conclusion, among lean or normal-weight adolescent patients with T1D inadequately controlled with insulin, metformin add-on therapy increased HRV despite no obvious effect on improvement in metabolic parameters, suggesting that metformin might have beneficial effects on cardiovascular autonomic function in this population. Further work is needed to evaluate whether metformin has the same effect on obese or adult patients with T1D. And long-term outcome trials assessing the effect of metformin on diabetes complications are of clinical importance and worthy to be conducted.

## Figures and Tables

**Table 1:** Baseline characteristics of the randomized participants.

Characteristic	Overall	Sequence A†	Sequence B†	*P* value
Number of subjects (*n*)	17	9	8	
Female, *n* (%)	7 (41.18)	3(33.33)	4 (50)	0.64
Age (years)	14.4 ± 2.3	15.2 ± 2.3	13.4 ± 1.8	0.09
Duration of diabetes (years)	4.50 (3.58, 6.92)	4.67 (4, 10.5)	3.96 (3.04, 5.88)	0.34
Tanner stage‡	4 (2-5)	4 (2-5)	3 (2-4)	0.24
BMI (kg/m^2^)	18.17 ± 1.81	18.08 ± 1.74	18.26 ± 2.0	0.85
Systolic BP (mmHg)	109.4 ± 10.5	112.0 ± 8.6	106.4 ± 12.3	0.29
Diastolic BP (mmHg)	64.5 ± 6.4	65.8 ± 4.5	63.0 ± 8.1	0.39
Insulin dose (units/kg/day)	0.94 (0.89, 1.02)	0.96 (0.91, 1.02)	0.92 (0.85, 1.06)	0.85
Lipid profiles				
TC (mmol/L)	4.55 ± 1.15	4.56 ± 1.32	4.54 ± 1.03	0.97
LDL-C (mmol/L)	2.53 (2.13, 3.25)	2.24 (2.13, 3.26)	2.58 (2.13, 2.99)	0.92
HDL-C (mmol/L)	1.64 ± 0.36	1.56 ± 0.31	1.74 ± 0.42	0.34
TG (mmol/L)	0.61 (0.51, 0.76)	0.52 (0.47, 0.61)	0.73 (0.61, 0.82)	0.03
Fasting glucose (mmol/L)	8.91 ± 3.02	8.47 ± 3.33	9.40 ± 2.77	0.54
HbA1c (%)	9.0 (8.5, 9.4)	8.7 (8.4, 9.1)	9.3 (8.7, 9.6)	0.18
Insulin sensitivity index (lnIS)§	2.24 ± 0.18	2.19 ± 0.22	2.29 ± 0.14	0.32
CGM parameters				
*n*	16	9	7	
MBG (mmol/L)	9.5 ± 1.83	9.26 ± 1.79	9.82 ± 1.99	0.56
%CV	25.71 ± 8.0	24.79 ± 6.19	26.90 ± 10.29	0.62
MAGE (mmol/L)	6.56 ± 2.51	5.99 ± 1.97	7.21 ± 3.05	0.37
MODD (mmol/L)	2.51 ± 1.0	2.20 ± 0.90	2.92 ± 1.07	0.20
Holter parameters				
*n*	15	8	7	
SDNN (ms)	154 ± 31.72	161.13 ± 28.30	145.86 ± 35.61	0.37
SDANN (ms)	139.2 ± 37.31	146.88 ± 31.04	130.43 ± 44.23	0.41
RMSSD (ms)	48.2 ± 13.82	45.88 ± 11.92	50.86 ± 16.25	0.50
PNN50 (%)	18 ± 10.35	18.25 ± 8.86	17.71 ± 12.58	0.92
LogLF (ms^2^)	6.59 ± 0.39	6.65 ± 0.35	6.52 ± 0.45	0.55
LogHF (ms^2^)	6.04 ± 0.66	6.14 ± 0.48	5.92 ± 0.85	0.56
LF/HF	1.93 ± 0.89	1.83 ± 0.84	2.04 ± 1.0	0.66

Data are mean ± standard deviation (SD), median (interquartile range (IQR)), or counts (percentage) unless otherwise noted. †Sequence A, metformin nonmetformin; sequence B, nonmetformin metformin. ‡Tanner stage was presented as median (range). §lnIS = 4.64725 − 0.02032 × waist (cm) − 0.09779 × A1c (%) − 0.00235 × TG (mg/dL, to convert TG values from mmol/L to mg/dL, divide by 0.0113). BMI: body mass index; BP: blood pressure; TC: total cholesterol; LDL-C: low-density lipoprotein cholesterol; HDL-C: high-density lipoprotein cholesterol; TG: triglyceride; CGM: continuous glucose monitoring; MBG: mean blood glucose; %CV: coefficient of variability of glycemia; MAGE: mean amplitude of glycemic excursions; MODD: means of daily differences; HRV: heart rate variability; SDNN: standard deviation of all RR intervals; SDANN: standard deviation of all the 5-minute RR intervals; RMSSD: square root of the mean of the sum of squares of differences between adjacent RR intervals; PNN50: the percentage of the interval differences of successive RR intervals greater than 50 ms; LF: low frequency; HF: high frequency; LF/HF: low frequency/high frequency.

**Table 2:** Effects of metformin add-on insulin vs. insulin therapy alone on metabolic parameters, glucose variability, and heart rate variability.

	Nonmetformin		Metformin		Treatment effect			
	Before	After 24 weeks	Before	After 24 weeks	Non-metformin	Metformin	Difference (95% CI)	*P*
Metabolic parameters								
*n*	17	15	16	15	15	15		
HbA1c (%)	9.2 ± 1.7	9.0 ± 1.5	9.1 ± 1.7	9.5 ± 1.9	0.03 ± 0.80	0.25 ± 0.97	0.28 (-0.39, 0.95)	0.40
FPG (mmol/L)	9.0 ± 2.93	9.83 ± 3.39	9.20 ± 3.13	7.50 ± 3.24	0.56 ± 3.73	−1.65 ± 4.55	-2.66 (-5.68, 0.35)	0.08
SBP (mmHg)	109.4 ± 10.5	111.0 ± 9.2	110.3 ± 9.9	112.5 ± 9.6	1.6 ± 9.7	1.5 ± 6.5	0.2(-6.2, 6.5)	0.96
DBP (mmHg)	67.4 ± 8.1	65.9 ± 5.8	64.2 ± 4.78	62.7 ± 7.3	−1.9 ± 6.8	−1.8 ± 7.1	-0.3(-5.6, 5.0)	0.90
BMI (kg/m^2^)	18.20 ± 1.50	19.59 ± 1.51	18.75 ± 1.86	19.12 ± 1.81	1.05 ± 1.02	0.48 ± 1.42	-0.46 (-1.38, 0.47)	0.32
TC (mmol/L)	4.57 ± 0.82	4.52 ± 0.98	4.39 ± 1.08	4.57 ± 0.57	−0.06 ± 0.67	0.06 ± 0.78	0.16 (-0.91, 0.17)	0.56
LDL-C (mmol/L)	2.62 (2.21, 3.25)	2.42 (2.11, 2.93)	2.25 (2.04, 2.71)	2.59 (2.38, 3.11)	-0.06 (-0.39, 0.33)	0.02 (-0.03, 0.40)	–	0.16^∗^
HDL-C (mmol/L)	1.67 ± 0.35	1.62 ± 0.32	1.59 ± 0.33	1.67 ± 0.30	−0.07 ± 0.15	0.03 ± 0.26	0.11 (-0.05, 0.27)	0.16
TG (mmol/L)	069 (0.59, 0.77)	0.61 (0.50, 0.82)	0.55 (0.47, 0.68)	0.69 (0.58, 0.77)	-0.09 (-0.23, 0.15)	0.13 (-0.07, 0.18)	–	0.22^∗^
Insulin dose (units/kg/day)	1.0 ± 0.28	1.0 ± 0.20	1.0 ± 0.24	0.99 ± 0.29	0.02 ± 0.14	0.01 ± 0.13	-0.03 (-0.13, 0.08)	0.61
lnIS†	2.22 ± 0.18	2.15 ± 0.29	2.17 ± 0.28	2.15 ± 0.19	-0.03(-0.15,0.03)	-0.06 (-0.18, 0.06)	–	0.64^∗^
CGM parameters								
*n*	16	13	16	14	13	14		
MBG (mmol/L)	10.92 ± 2.77	10.71 ± 2.95	9.33 ± 1.75	11.22 ± 2.90	−0.45 ± 2.57	1.70 ± 3.09	1.82 (-0.53, 4.17)	0.12
%CV	24.50 ± 8.09	27.34 ± 5.56	26.45 ± 6.28	26.67 ± 9.20	4.29 ± 10.66	1.08 ± 11.55	-1.91(-11.14, 7.32)	0.67
MAGE	7.08 ± 2.55	6.33 ± 2.53	6.01 ± 1.98	6.96 ± 2.23	−0.79 ± 3.52	0.77 ± 1.87	1.67 (-1.12, 4.45)	0.23
MODD	3.43 ± 1.40	3.52 ± 1.02	2.91 ± 1.19	3.78 ± 1.40	0.29 ± 1.27	0.99 ± 1.36	0.49 (-0.66, 1.64)	0.38
Holter parameters								
*n*	15	14	15	14	14	14		
SDNN (ms)	160.93 ± 47.56	162.15 ± 38.13	157.29 ± 27.79	175.1 ± 48.82	−4.75 ± 20.76	19.50 ± 31.07	26.96 (2.24, 51.69)	0.03
SDANN (ms)	146.07 ± 50.81	144.92 ± 42.14	140.29 ± 31.84	160.2 ± 48.19	−9 ± 19.80	16.6 ± 31.46	25.62 (0.15, 51.09)	0.049
RMSSD (ms)	54.29 ± 20.06	53.15 ± 29.40	43.43 ± 13.08	57.40 ± 22.53	−3.67 ± 25.85	13.0 ± 15.63	17.90 (-3.08, 38.90)	0.09
PNN50	19.93 ± 12.11	15.15 ± 9.39	17.29 ± 9.12	21.6 ± 11.33	−4.83 ± 8.64	4.5 ± 5.60	10.14 (3.74, 16.55)	0.004
LogLF (m^2^)	6.53 ± 0.45	6.46 ± 0.40	6.58 ± 0.42	6.61 ± 0.45	−0.07 ± 0.25	−0.06 ± 0.29	0.08 (-0.15, 0.31)	0.46
LogHF (m^2^)	6.00 ± 0.71	5.87 ± 0.60	6.04 ± 0.62	6.17 ± 0.65	−0.19 ± 0.34	0.01 ± 0.35	0.38 (0.09, 0.66)	0.01
LF/HF	1.87 ± 0.83	1.91 ± 0.64	1.85 ± 0.77	1.81 ± 0.66	0.16 ± 0.36	−0.27 ± 0.26	-0.47 (-0.75, -0.18)	0.006

Estimated treatment effect difference and *P* value were derived from linear mixed effects models, with treatment stage, carryover, sequence, and treatment as fixed effects and study participant and sequence as nested random effects, unless otherwise noted. ^∗^*P* value was derived from the Wilcoxon signed rank test. †lnIS = 4.64725 − 0.02032 × waist (cm) − 0.09779 × A1c (%) − 0.00235 × TG (mg/dL, to convert TG values from mmol/L to mg/dL, divide by 0.0113). BMI: body mass index; BP: blood pressure; TC: total cholesterol; LDL-C: low-density lipoprotein cholesterol; HDL-C: high-density lipoprotein cholesterol; TG: triglyceride; CGM: continuous glucose monitoring; MBG: mean blood glucose; %CV: coefficient of variability of glycemia; MAGE: mean amplitude of glycemic excursions; MODD: means of daily differences; HRV: heart rate variability; SDNN: standard deviation of all RR intervals; SDANN: standard deviation of all the 5-minute RR intervals; RMSSD: square root of the mean of the sum of squares of differences between adjacent RR intervals; PNN50: the percentage of the interval differences of successive RR intervals greater than 50 ms; LF: low frequency; HF: high frequency; LF/HF: low frequency/high frequency.

**Table 3:** Linear regression analyses for the association between metformin add-on treatment and changes in HRV indexes in youth with type 1 diabetes.

Model	Dependent variable	*β*-Coefficient	95% CI	*P*
Unadjusted model	*Δ*SDNN	24.25	1.10 to 47.39	0.04
	*Δ*SDANN	25.60	2.64 to 48.56	0.03
	*Δ*PNN50	9.33	2.70 to 15.97	<0.01
Multivariable model†	*Δ*SDNN	28.62	1.67 to 55.58	0.04
	*Δ*SDANN	31.0	2.76 to 59.24	0.03
	*Δ*PNN50	9.63	3.56 to 15.73	<0.01

All models with metformin treatment as independent variable. †Multivariable model with adjustment for changes in HbA1c, systolic blood pressure, and mean amplitude of glycemic excursions (MAGE). HRV: heart rate variability; CI: confidence interval; SDNN: standard deviation of all RR intervals; SDANN: standard deviation of all the 5-minute RR intervals; PNN50: the percentage of the interval differences of successive RR intervals greater than 50 ms.

## Data Availability

The data used to support the findings of this study are available from the corresponding author upon request.
